# Ectopic Pregnancy in an Adolescent: A Case Report and Review of Literature

**DOI:** 10.7759/cureus.32220

**Published:** 2022-12-05

**Authors:** Elham Neisani Samani, Melina M Henderson, Hamid Sanjaghsaz, Ronald Nichols

**Affiliations:** 1 Obstetrics and Gynecology, Garden City Hospital, Garden City, USA; 2 Obstetrics and Gynecology, Michigan State University, Garden City, USA

**Keywords:** tubal ectopic pregnancy, ectopic pregnancy, methotrexate, discriminatory human gonadotropin zone, adolescent

## Abstract

Ectopic pregnancy continues to be the leading cause of death in the first trimester. Here, we report the case of a 17-year-old female who presented with vaginal bleeding and a positive serum beta-human chorionic gonadotropin level. In addition, we review the literature, focusing on the early diagnosis and management with the increasing preference for nonsurgical management of ectopic pregnancy, particularly in adolescents.

## Introduction

The blastocyst normally implants in the endometrial lining of the uterine cavity. Implantation anywhere else is defined as an ectopic pregnancy [1,2]. The incidence of ectopic pregnancy is difficult to estimate because inpatient hospital treatment of ectopic pregnancy has decreased and multiple healthcare visits for a single ectopic pregnancy have increased. In a study using data from emergency departments, the incidence of ectopic pregnancy from 2006 to 2013 increased from 11 to 13.7 ectopic pregnancies per 1,000 live births and from 7% to 8.3% ectopic pregnancy per 1,000 pregnancies [3,4]. Ectopic pregnancy remains a life-threatening emergency in early pregnancy leading to 10% of maternal deaths in the United States [4,5]. Although the true prevalence is unknown, approximately 2% of pregnancies are reported as ectopic pregnancies [1,2]. Further, 95% of ectopic pregnancies occur in the fallopian tubes; however, it also occurs in the cervix, ovary, hysterotomy scar, and abdomen [2-4]. Risk factors for ectopic pregnancy include but are not limited to age more than 35 years, history of prior ectopic pregnancy, history of pelvic infection or surgeries, smoking, intrauterine device (IUD), age at first intercourse less than 18 years old, and sexually transmitted infections including gonorrhea and chlamydia [4-6]. The most common presenting signs of ectopic pregnancy are pelvic pain and vaginal bleeding [1-3]. Ectopic pregnancy can be treated medically and surgically [3-6]. We present a case of ectopic pregnancy in a teenager, which was diagnosed by monitoring human chorionic gonadotropin (hCG) and ultrasound scans. Further management with methotrexate administration was undertaken which successfully treated the ectopic pregnancy.

## Case presentation

A 17-year-old, nulliparous patient with no significant medical history presented to the emergency department (ED) complaining of lower abdominal pain and vaginal spotting. Her last menstrual period was seven weeks prior to her ED visit to our facility. The patient complained of three days of progressive severe abdominal pain in her left lower quadrant. She admitted to being in a monogamous relationship with a male partner. The patient denied any history of sexually transmitted infections, pelvic infection disease, or intrauterine device (IUD) use. She denied smoking. She also denied any history of abdominal or pelvic surgeries.

The electronic medical record was reviewed via Epic, in which “care everywhere” was utilized, allowing access to records at another healthcare facility. The patient had been seen three times in another facility, where her provider performed a serum hCG level and transvaginal ultrasound. It was documented that the patient had an hCG of 4,788 mIU/mL at that time. In addition, her transvaginal ultrasound revealed no evidence of intrauterine pregnancy, with a complex mass of the left ovary measuring 2.1 cm, along with a moderate amount of free fluid in the pelvis. At the other facility, she was expectantly managed and observed overnight with serial abdominal examinations and repeat hCG levels. They discharged her in stable condition and instructed her to follow up as an outpatient in 48 hours to check the hCG level. The patient came to our ED on the same day as her discharge from another facility. When she visited our ED for the same complaints, her serum hCG was 2,790 mIU/mL and 2,144 mIU/mL in 24 and 48 hours, respectively, after the first measurement.

In our ED, she was normotensive at 118/67 mmHg, with a pulse rate of 96 beats per minute, respiratory rate of 16 breaths per minute, and temperature of 98.1°F. Her examination was significant for tenderness to palpation of her left lower quadrant abdomen, without rebound or guarding. She was hemodynamically stable with a hemoglobin of 12.1 g/dL along with a white blood count of 5.20 mm^3^. A vaginal examination revealed moderate bleeding. Upon arrival at the ED, the patient passed some clots with tissues which were sent to pathology.

**Figure 1:**
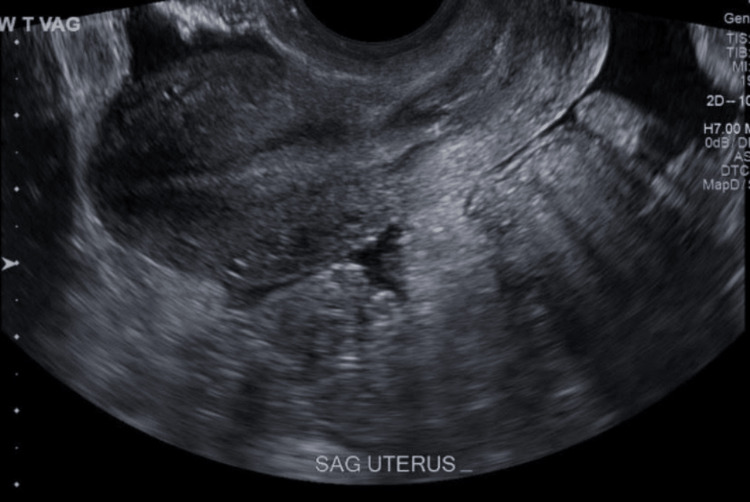
Transvaginal ultrasonogram. The longitudinal image revealed the uterine cavity with no intrauterine pregnancy.

**Figure 2:**
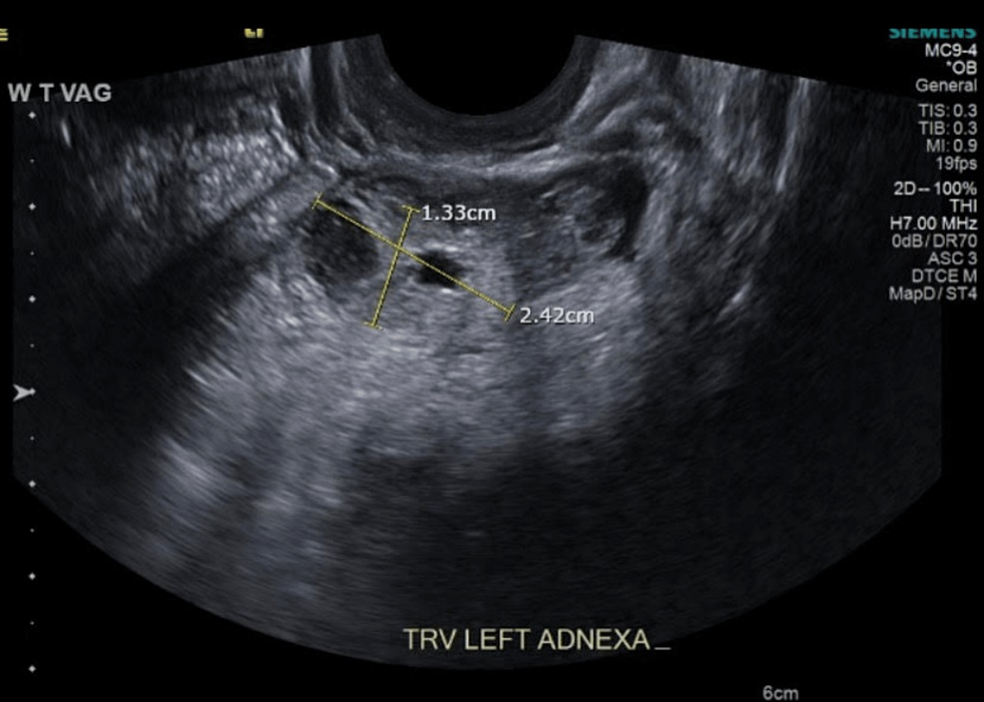
Transvaginal ultrasonogram. The sagittal image shows the complex area measuring 24 × 13 mm in the left ovary.

During this presentation, hCG was 1,650 mIU/mL. Her alanine aminotransferase and aspartate aminotransferase were within normal limits. The transvaginal ultrasound did not reveal an intrauterine pregnancy. In the left adnexa adjacent to the left ovary, a complex area measuring 24 × 13 mm was identified, suspicious for ectopic gestation (Figures 1, 2). The histologic examination of the intrauterine material confirmed the diagnosis of decidual tissue, without trophoblasts, or any identifiable chorionic villi.

The patient was found to have a positive genital culture for chlamydia and was treated for it with azithromycin. After counseling and consent, medical management with one dose of methotrexate was undertaken. The patient was administered a single systemic dose of methotrexate at 50 mg/m^2^ body surface area. Serum hCG on day four was 607 mIU/mL. The patient was followed as an outpatient until the resolution of her serum hCG to a nonpregnant level.

## Discussion

Ectopic pregnancy is a life and fertility-threatening condition that is still a major concern for women of reproductive age [2,3]. The etiology of ectopic pregnancy remains uncertain. Although several risk factors including tobacco smoking, multiple sexual partners, previous ectopic pregnancy, damage to the fallopian tubes from pelvic inflammatory disease, or previous surgery have been identified, the diagnosis can be challenging, relying on a combination of serial serum hCG measurements and ultrasound scanning. Women with clinical signs and symptoms of a ruptured ectopic pregnancy including hemodynamic instability or an acute abdomen should be treated urgently. Current therapeutic options for ectopic pregnancy are medical and surgical therapy. Most cases of ectopic pregnancy which are detected early can be treated successfully either with medical management using methotrexate or with minimally invasive surgery [4-7].

During the past 50 years, many studies have been performed to determine the discriminatory hCG level. Kader et al. recommended an hCG level of 6,500 mIU/mL in 1981. With the introduction of transvaginal ultrasonography, the gestational sac became detectable earlier in pregnancy, and the reported discriminatory hCG level was reduced to 2,000 mIU/mL [8,9]. According to the American College of Obstetricians and Gynecologists (2018), the discriminatory hCG level should be as high as 3,500 mIU/mL to avoid misdiagnosis and possible interruption of an intrauterine pregnancy. The discriminatory level refers to an hCG level above which the landmarks of a normal intrauterine gestation should be visible on ultrasonography. The absence of a possible gestational sac on ultrasound in the presence of an hCG measurement above the discriminatory level strongly suggests a nonviable gestation such as an early pregnancy loss or an ectopic pregnancy [9,10]. In our case, transvaginal ultrasound showed no evidence of an intrauterine pregnancy with a serum hCG of 4,788 mIU/mL. We believe the serum hCG values should be correlated with the patient’s history, clinical presentation, and ultrasound findings. In addition, histologic examination of the intrauterine material confirmed the diagnosis of decidual tissue, without trophoblasts, and no identifiable chorionic villi, which supported an ectopic pregnancy as a definitive diagnosis. The choice of methotrexate single-dose protocol was most appropriate for this patient, with a relative plateau of her hCG values [5].

We wish to draw attention to the medical management of ectopic pregnancy in adolescents. Adolescents would benefit from early diagnosis and appropriate medical treatment. Any patient who presents with amenorrhea, pain, or vaginal bleeding should be evaluated for a possible ectopic pregnancy. A transvaginal ultrasound should be performed, and if an intrauterine pregnancy or ectopic pregnancy is not seen, a correlation with serum hCG levels should be done.

Providers must routinely screen adolescents for and treat sexually transmitted diseases such as chlamydia to decrease the risk of tubal damage and ectopic pregnancy. Unruptured ectopic pregnancy may be treated with expectant management, medical management (methotrexate), and surgical management. Healthy teens with unruptured ectopic pregnancies who are able and willing to undergo close surveillance may be treated with methotrexate.

## Conclusions

Our case illustrates that the serum hCG values should be correlated with the patient’s history, clinical presentation, and ultrasound findings. Once a diagnosis of ectopic pregnancy has been made, the therapeutic options of medical management and surgical intervention can be offered. While surgical management is the most effective and medical management is more effective than expectant management, all are acceptable options when the patient has been appropriately counseled.
